# Risk Factors for Progressive Visual Field Loss in Primary Angle-Closure Glaucoma: A Retrospective Cohort Study

**DOI:** 10.1371/journal.pone.0069772

**Published:** 2013-07-08

**Authors:** Nai-Wen Fan, De-Kuang Hwang, Yu-Chieh Ko, Fan-Chen Tseng, Kuo-Hsuan Hung, Catherine Jui-Ling Liu

**Affiliations:** 1 Department of Ophthalmology, Taipei Veterans General Hospital, Taipei, Taiwan; 2 Institute of Clinical Medicine, National Yang Ming University, Taipei, Taiwan; 3 Department of Ophthalmology, Taoyuan Veterans Hospital, Taoyuan, Taiwan; 4 Department of Public Health and Institute of Public Health, National Yang-Ming University, Taipei, Taiwan; 5 National Yang-Ming University School of Medicine, Taipei, Taiwan; 6 National Institute of Infectious Diseases and Vaccinology, National Health Research Institutes, Tainan, Taiwan; 7 Department of Epidemiology, University of North Carolina at Chapel Hill, Chapel Hill, North Carolina, United States of America; 8 Department of Ophthalmology, National Yang-Ming University Hospital, Yilan, Taiwan

## Abstract

**Purpose:**

To investigate risk factors associated with progressive visual field (VF) loss in primary angle closure glaucoma (PACG).

**Methods:**

We retrospectively reviewed medical record of PACG patients who had ≥5 reliable VF examinations (central 24-2 threshold test, Humphrey Field Analyzer) and ≥2 years of follow-up. Each VF was scored using Collaborative Initial Glaucoma Treatment Study system. Progression was defined if 3 consecutive follow-up VF tests had an increased score of ≥3 above the mean of the first 2 VF scores. Factors associated with VF progression were evaluated by Cox proportional hazards models.

**Results:**

A total of 89 eyes from 89 patients (mean age, 69.8 ± 7.9 years), who received a mean of 6.9 ± 2.3 VF tests (mean deviation at initial, -8.1 ± 4.4 dB) with a mean follow-up of 63.9 ± 23.9 months were included. VF progression was detected in 9 eyes (10%). The axial length (AL), anterior chamber depth, and intraocular pressure (IOP) in patients with and without progression were 22.5 ± 0.6 and 23.1 ± 0.9 mm, 2.5 ± 0.3 and 2.5 ± 0.3 mm, 14.8 ± 2.4 and 14.3 ± 2.3 mm Hg, respectively. AL was the only factor associated with progression in both Cox proportional hazards univariate (p = 0.031) and multivariate models (p = 0.023).

**Conclusion:**

When taking into account age, IOP, follow-up period, and number of VF tests, a shorter AL is the only factor associated with VF progression in this cohort of Chinese patients with PACG. Further studies are warranted to verify the role of AL in progressive VF loss in PACG.

## Introduction

Glaucoma is the second leading cause of blindness worldwide [1,2]. The proportion of blindness caused by angle closure glaucoma (ACG) is greater than that caused by open angle glaucoma (OAG) [1,3]. Quigley et al estimated that the number of people with bilateral blindness from ACG will be 5.3 million by 2020 [3]. More than 80% of ACG patients resides in Asia, and around 50% lives in China [3].

The well-known risks factors for visual field (VF) progression in glaucoma were learned mostly from studies on OAG, including Collaborative Initial Glaucoma Treatment Study (CIGTS) [4], Collaborative Normal Tension Glaucoma Study [5], Advanced Glaucoma Intervention Study (AGIS) [6], and Early Manifest Glaucoma Trial (EMGT) [7]. The identified risk factors include: older age [8], higher baseline intraocular pressure (IOP) [8], higher IOP during the follow-up period [5,6,9] thinner central corneal thickness (CCT) [8], self-reported diabetes mellitus [4,10], more severe VF mean deviation (MD) at baseline [9], lower blood pressure [8], presence of migraine [11], presence of disc hemorrhage [8,9,11], and wider IOP fluctuation [12]. Only few studies have assessed risk factors associated with disease progression in primary ACG (PACG). Hong et al reported chronic PACG patients with thinner CCT were at risk of VF progression by comparing the final and earliest VF MD [13]. Since MD is subject to many factors such as test variability and lens opacity, comparing MD between two time points may not reflect true glaucoma progression. Another retrospective study conducted by the same group found that long-term IOP fluctuation was associated with progressive VF deterioration in PACG [14]. According to Quek et al, neither mean IOP nor IOP range was a risk factor of VF progression in PACG without prior acute angle closure (AAC) [15]. Therefore, risk factors for VF progression in PACG remain elusive.

To determine risk factors of disease progression is crucial in the management of PACG and prevention of blindness. Thus, the purpose of this study was to investigate the rate of and risk factors associated with VF progression in Chinese patients with PACG.

## Methods

This retrospective study reviewed medical record of all patients who met the International Classification of Disease, 9th revision, Clinical Modification (ICD-9-CM): 365.23, chronic angle closure glaucoma in 2008 at Taipei Veterans General Hospital, a tertiary referral hospital. Data of each patient were collected since the date when the first of the two reliable baseline VF tests was accomplished. The Institutional Review Board (IRB) for Human Research at the Taipei Veterans General Hospital approved our review of patient records in this study. Informed written consent was waived by the approving IRB.

Patients were included if they had received regular follow-up (every 1–4 months) at glaucoma service for at least 2 years with 5 or more reliable VF tests (≤33% fixation losses, ≤33% false-negative results, and ≤33% false-positive results). All subjects met the following inclusion criteria: eyes with an occludable angle; glaucomatous optic neuropathy with corresponding VF defects; and a best-corrected visual acuity (BCVA) of 20/100 or better throughout the study period. An occludable angle was defined if the posterior trabecular meshwork was invisible on gonioscopy for at least 270° of the angle circumference in the primary position without indentation [16]. Glaucomatous optic disc was defined as a vertical cup to disc ratio (VCDR) of the optic nerve head ≥0.7, a VCDR difference of ≥0.2 between eyes, or focal thinning, notching, or excavation of the neuroretinal rim. VF tests were conducted with a Humphrey Field Analyzer set for the central 24-2 threshold test with size III white stimulus (Carl Zeiss Meditec, Inc, Dublin, California, USA). Abnormal VF tests were defined as outside normal limits of glaucoma hemifield test or pattern standard deviation (PSD) outside the 95% normal limits.

Patients with the following conditions were excluded: secondary angle closure glaucoma attributed to medicine or other ocular abnormalities; history of ocular surgery other than cataract or glaucoma surgery; corneal pathologic features; and other retinal or neurologic diseases that could possibly be associated with VF progression. This study used the CIGTS scoring system to identify VF progression. Patients with a mean CIGTS score of the first 2 VFs that exceeded 17.0 were excluded. If both eyes of one patient were eligible, the eye with the worse VF MD was included in the study.

Data recorded included patient demographics, past history, personal history, family history, systemic diseases, and ophthalmic findings. Axial length (AL), anterior chamber depth (ACD), and lens thickness of the phakic eye were measured by a contact ultrasound biometry (A-scan, Sonomed Inc., USA). If an A-scan had not been performed, AL and ACD measured by IOL Master (Version 5.02; Carl Zeiss Meditec Ltd, Jena, Germany) were recorded. For patients who were pseudophakic at enrollment into this study, only AL was recorded. CCT was measured by ultrasonic pachymetry. All IOP measurements before VF progression were recorded. To avoid short-term fluctuations in IOP that resulted from operation or intervention, we disregarded the IOP within one month after operation or needling of the bleb and within one week after laser treatment. The mean IOP represented the mean of all recorded IOP values. Highest IOP (peak IOP) was also recorded. Standard deviation of the IOP was used as a surrogate for IOP fluctuation. The IOP range represented the value of the highest IOP minus the lowest IOP during the study period.

### Progression of VF loss

The VF score was generated from the total deviation probability plot values on the Humphrey 24-2 printout based on the methods developed in CIGTS [17]. In brief, each of the 52 points was deemed as a point of defect if its probability value was 0.05 or less. A weight was assigned depending on the minimum depth of the defect at the given point and its 2 most defective neighboring points in the same hemifield, which ranged from 1 to 4. The weights for all 52 points were summed, then divided by 10.4 to obtain a value between 0 (normal) and 20 (perimetrically blind) [17].

Evidence of progression was declared if the overall CIGTS VF score increased by 3 or more above the mean of the first 2 VF scores on 3 consecutive tests at different visits.

### Statistical analysis

Independent samples *t*-test was used to compare continuous variables between patients with and without progression and the Pearson Chi-Square was used for categorical variables comparison. Hazard ratios (HRs) for the association between risk factors and progression of VF defects (yes or no) were obtained using Cox proportional hazards models based on the follow-up time until progression. Variables with a p < 0.2 in the univariate model were further analyzed in a multivariate model. We report adjusted HRs from multivariate models as well as the 95% confidence interval (CI) for each risk factor. p < 0.05 was considered statistically significant.

## Results

Overall, 1598 patients with ICD-9-CM: 365.23, chronic angle closure glaucoma visited our department in 2008. Among them, eighty-nine eyes of 89 patients conformed to all the inclusion and exclusion criteria. All of the patients were of Chinese ethnicity, with a mean age of 69.8 ± 7.9 years at enrollment. Six eyes (7%) had a history of prior AAC. Eighty-seven (98%) patients had received laser peripheral iridotomy (LPI), and 2 (2%) patients without LPI had undergone combination surgery consisting of cataract extraction and trabeculectomy previously. Seventy-seven eyes (87%) were phakic at baseline. The average MD of the first VF was -8.1 ± 4.4 dB and the mean PSD was 6.9 ± 4.0 dB. The mean IOP during the study period was 14.4 ± 2.3 mm Hg. The mean number of VF tests collected was 6.9 ± 2.3 over a mean follow-up time of 63.9 ± 23.9 months, ranging from 24.4 to 134.0 months, with 84 (94%) patients followed up for more than 3 years.

During the follow-up period, 9 (10%) patients developed VF progression. Demographic and clinical characteristics of these 2 groups are shown in Table 1. There was no statistical difference between the 2 groups for any parameter (Table 1). Only 4 patients had ACD and AL obtained with IOL Master, and all of these patients were in the non-progression group.

**Table 1:** Comparison of clinical characteristics between primary angle closure glaucoma with and without visual field progression.

**Parameter**	**Visual field progression (n = 9)**	**No visual field progression (n = 80)**	**p value**
Age, year	71.5 ± 6.5	69.6 ± 8.0	0.481^a^
Female sex, %	2 (22)	33 (41)	0.262^b^
Follow-up, month	65.8 ± 33.0	63.7 ± 23.0	0.809^a^
Diabetes mellitus, %	2 (22)	11 (14)	0.474^b^
Hypertension, %	5 (56)	35 (44)	0.505^b^
Smoker, %	0 (0)	6 (8)	0.401^b^
Positive family history of glaucoma, %	0 (0)	4 (5)	0.498^b^
Previous acute angle closure, %	1 (11)	5 (6)	0.685^b^
Phakic eyes at baseline, %	9 (100)	68 (85)	0.218^b^
Baseline IOP, mm Hg	16.2 ± 2.9	15.8 ± 3.7	0.732^a^
Baseline VF			
pattern standard deviation, dB	7.1 ± 4.6	6.8 ± 4.0	0.834^a^
mean deviation, dB	-7.8 ± 4.7	-8.2 ± 4.4	0.795^a^
Central corneal thickness, μm	530.7 ± 39.2	548.4 ± 35.0	0.159^a^
Anterior chamber depth, mm	2.5 ± 0.3 (n = 8)	2.5 ± 0.3 (n = 59)	0.923^a^
Lens thickness, mm	4.9 ± 0.5 (n = 8)	4.8 ± 0.6 (n = 54)	0.685^a^
Axial length, mm	22.5 ± 0.6 (n = 8)	23.1 ± 0.9 (n = 68)	0.074^a^
No. of VF examinations	6.8 ± 2.0	6.9 ± 2.4	0.846^a^
IOP, mm Hg	14.8 ± 2.4	14.3 ± 2.3	0.605^a^
IOP fluctuation, mm Hg	2.8 ± 0.9	2.2 ± 0.9	0.123^a^
IOP range, mm Hg	9.3 ± 2.3	10.8 ± 2.3	0.090^a^
Peak IOP, mm Hg	21.7 ± 5.4	20.2 ± 5.3	0.415^a^
Disc hemorrhage during study period, %	1 (11)	6 (8)	0.685^b^
Surgery during study periods			
Trabeculectomy, %	1 (11)	8 (10)	0.474^b^
Cataract surgery, %	3 (33)	18 (23)	0.267^b^
Cataract and trabeculectomy, %	1 (11)	2 (3)	0.167^b^
Laser treatment during study periods			0.327^b^
Gonioplasty, %	0 (0)	2 (3)	
ALTP, %	0 (0)	6 (8)	

ALTP, argon laser trabeculoplasty; IOP, intraocular pressure; VF, visual field ^a^ Independent samples t test; ^b^ Pearson Chi-Square

Cox proportional hazards univariate analysis tested all of the potentially relevant variables listed in Table 1. Only CCT and AL were associated with VF progression at p < 0.2. The univariate HR of CCT was 0.98 per μm thicker, 95% CI, 0.96 to 100 (p = 0.111). The univariate HR of AL was 0.35 per mm longer, 95% CI, 0.13 to 0.91 (p = 0.031). Neither mean IOP nor peak IOP was a risk factor for VF progression (p = 0.914 and 0.988, respectively). Likewise, neither IOP fluctuation nor IOP range was relevant to VF progression (p = 0.566 and 0.561, respectively). Given that age and gender are risk factors for PAC, a multivariate analysis was performed with these 2 variables in addition to CCT and AL. It turned out that a longer AL was a protective factor (adjusted HR, 0.28 per mm longer; 95% CI, 0.09 to 0.84; p = 0.023) (Table 2). If we excluded AL data obtained with IOL Master, the AL variable still remained significant (adjusted HR, 0.24 per mm longer; 95% CI, 0.07 to 0.78; p = 0.017).

**Table 2:** Cox proportional hazards multivariate analysis testing the association between age, gender, axial length, central corneal thickness, and the hazards of visual field progression.

**Parameter**	**Hazard ratio**	**95% confidence interval**	**p value**
Age (per year older)	0.98	0.85 to 1.13	0.814
Gender (female)	8.00	0.80 to 79.92	0.077
Axial length (per mm longer)	0.23	0.09 to 0.84	0.023
Central corneal thickness (per μm thicker)	0.99	0.96 to 1.02	0.443

Four of the nine patients with VF progression underwent cataract surgery during the study period; they received cataract surgery at least 1 year before VF progression was identified. The mean interval between cataract operation and progression was 30.0 ± 12.1 months, ranging from 12.3 to 43.5 months. In the other five phakic eyes with VF progression, there was a mean drop of 1.4 lines of BCVA at progression compared to that at baseline (range, -3 to +2 lines; final BCVA range, 0.5 to 1.0).

## Discussion

This study showed that a shorter AL was a risk factor for progressive VF defects in Chinese patients under treatment for PACG. To the best of our knowledge, this finding has never been previously reported. To date, a standard criterion to define VF progression is still lacking. However, the concensus is to use event-based methods in the first few years of follow-up when multiple VF tests are not available for an authentic trend analysis. We adopted the CIGTS criteria to define VF progression because it has been shown to have good specificity and the best sustainablity as compared to other VF progression algorithms [18].

Quek et al have reported that higher mean IOP and the presence of previous AAC were associated with progression of VF defects in a study of Chinese patients with PACG [15]. However, neither of these factors was identified as risk factors for VF progression in our study. The discrepancy in study results may be due to differences in the definition of VF progression; AGIS system was used in Quek et al’s report and CIGTS system in our study. Another possible explanation is that the number of eyes (7%) with previous AAC was small in our study compared with that (35%) in the study by Quek et al [15]. In fact, if subjects with prior AAC were excluded, they found mean IOP and the range of IOP were no longer associated with VF progression. Quek et al reasoned that mean IOP probably was higher in eyes with prior AAC due to the acute pressure spikes and the difficulty in defining the end of acute episodes [19].

The finding that a shorter AL was associated with disease progression in treated PACG may be explained by a greater circadian change in habitual IOP in those eyes with a shorter AL. Loewen et al reported that a shorter AL was significantly associated with greater posture-dependent changes in IOP [20]. Other studies also found choroidal thickness was thicker in eyes with a shorter AL and the increase in choroidal thickness after water drinking was greater in eyes with angle closure than in eyes with open angle [21,22]. Accordingly, it is likely that eyes with a shorter AL are prone to greater fluctuations of circadian IOP related to variations in physiology, posture, and environmental stimulation, which contribute to further damage of the optic nerve. In fact, in a study with a mean follow-up of 9 years, higher IOP fluctuation was associated with faster VF progression in PACG patients whose IOP was maintained below 18 mm Hg after trabeculectomy and phacoemulsification [14]. In our study, the mean IOP fluctuation was higher in patients with VF progression than in those without progression, albeit not reaching statistical significance. It is worth noting that the so-called IOP fluctuations in almost all clinical studies may not reflect the genuine circadian fluctuations of pressure that act on the optic nerve head and retinal nerve fiber. Despite the introduction of devices allegedly being able to measure IOP in a continuous way without interrupting sleep at night, the accuracy and feasibility of them remain to be verified [23].

ACD was not associated with VF progression in both Quek et al’s report [15] and the present study. These results substantiate that central ACD is not a crucial parameter for Chinese patients with PACG [24,25]. Nolan et al and Wang et al have suggested that angle closure in Chinese was mainly attributable to crowding of peripheral anterior chamber, plateau iris configuration, or a combination of these features together with pupil block, rather than pure pupil block [25,26].

Because of the strict criteria for inclusion and exclusion, only 89 eyes constituted this study and nine of them showed progression. In addition to the small sample size, the limitations of our study are related to the inherent bias of its retrospective nature. Data of post-iridotomy gonioscopic finding is incomplete. The AL measurements of 4 patients were obtained using IOL Master instead of A-scan biometry. AL measurements using IOL Master was longer than that obtained with ultrasound biometry [27]. However, AL remained a significant risk factor in multivariate analysis even when we excluded these 4 patients. This study did not exclude patients with coexistent cataract, so VF deterioration might be caused by increasing severity of lens opacity. However, the BCVA decreased slightly (< 2 lines) during the study period for the 5 eyes that remained phakic and showed VF progression. Besides, VF progression was detected long after cataract surgery for the 4 eyes that underwent cataract extraction during the study period. This suggested that increasing lens opacity was unlikely to be responsible for VF progression in our study.

This study demonstrates that a shorter AL is the only risk factor for VF progression in PACG. With a larger sample size, more risk factors associated with progression might be able to be identified since small sample size creates a limitation in performing multivariable analysis. Further prospective studies using ultrasound biomicroscopy or anterior segment optical coherence tomography are warranted to validate the biological impact of AL on dynamic changes of the anterior chamber angle and its possible role in PACG progression. Although IOP was not identified as a risk factor, it must be noted that all patients included in this study were treated by glaucoma specialists with controlled IOP. Thus, the results of our study are not applicable to PACG eyes with uncontrolled IOP. 
